# ImmunoFISH Is a Reliable Technique for the Assessment of 1p and 19q Status in Oligodendrogliomas

**DOI:** 10.1371/journal.pone.0100342

**Published:** 2014-06-20

**Authors:** Céline Duval, Marie de Tayrac, François Sanschagrin, Karine Michaud, Peter Vincent Gould, Stéphan Saikali

**Affiliations:** 1 Department of Pathology, Centre Hospitalier Universitaire de Québec, Québec, Canada; 2 Department of genomic and molecular genetics, Centre Hospitalier Universitaire de Rennes, Rennes, France; 3 Department of Neurosurgery, Centre Hospitalier Universitaire de Québec, Québec, Canada; University of Navarra, Spain

## Abstract

**Objective:**

To develop a new ImmunoFISH technique for the study of oligodendrogliomas by combining a standard immunohistochemical stain using MIB-1 antibody with a standard FISH technique using commercial 1p36 and 19q13 chromosomal probes.

**Methods:**

Validation was performed by two observers on a series of 36 pre-selected oligodendrogliomas and compared to the results previously determined by FISH alone.

**Results:**

The ImFISH technique is easy to perform and to analyze and is no more time-consuming than the usual FISH technique. Our results show that the inter-observer reliability of ImFISH is high (κ = 0.86 and 0.95 respectively for 1p and 19q). Compared to FISH, the ImFISH exhibits a very high sensitivity (∼100%) and specificity (∼90%) for 1p and/or 19q deleted cases. The sensitivity is high for normal cases (∼85%) and imbalanced cases (∼90%) with a specificity ranging between 50 and 85%. Finally, there were no significant differences between FISH and ImFISH results calculated on 60, 40 or 20 cells.

**Conclusion:**

Our study demonstrates the reliability of the ImFISH technique in oligodendrogliomas and emphasizes its advantage in poorly cellular tumoral specimen.

## Introduction

The study of chromosome 1p and 19q status has become an essential step in the treatment of oligodendroglial tumors in recent years [1], [2], [3], [4], [5], [6], [7], [8], [9], [10], [11], [12]. Codeletion of 1p and 19q whole arms is strongly correlated with a better response to standard treatment with radiotherapy and chemotherapy as well as a better overall survival [13], [14], [15], [16], [17], [18], [19], [20], [21].

Several molecular techniques are described in the literature to study the chromosomal status of tumor cells, including fluorescence in situ hybridization (FISH) [22], [23], [24], [25], [26], polymerase chain reaction [27], [28], quantitative microsatellite analysis [14], [29], loss of heterozygosity (LOH) by microsatellite analysis [8], [10], [13], [30], [31], [32], [33], [34], [35], comparative genomic hybridization array (CGH) [17], [36], [37], [38], [39], [40], [41] and multiplex ligation - dependent probe [42]. All these techniques have their advantages and disadvantages but the most widely used among them is FISH [26] because it can be performed by fluorescent microscopy on paraffin embedded tumor tissue sections and is thus easily accessible to most pathology laboratories.

Although some guidelines exist in the literature to harmonize the interpretation of FISH results [43], [44], [45], several authors have emphasized the difficulty that may be encountered in the interpretation of chromosomal signals, especially in polyploid cases [26], [46], [47]. The main causes of these difficulties include the thickness of the histological section, a low density of tumor cells in some specimens or conversely a high density of tumor cells with overlapping nuclear profiles, making their FISH interpretation difficult [26], [47].

To optimize the detection of chromosomal status by FISH technique, some authors have proposed to replace the standard histological section from paraffin-embedded tissue with tumor cell nuclei isolated from paraffin embedded blocks [46], frozen smears [48] or fresh tissue touch preparations [49]. Other authors have proposed to sample a larger number of tumor cells by using automatic analysis [44] or to perform a chromogenic technique using dual-color chromogenic in situ hybridization (CISH) [50].

Another technique proposed to increase the diagnostic yield of FISH involves adding an immunochemical step to permit simultaneous analysis of the genotype and the phenotype on the same tissue sample. This technique called ImmunoFISH (ImFISH) was first described in the literature in the 90 s and was originally developed for the study of hematologic malignancies [51], [52], [53]. Its use has since been extended to other neoplastic processes including those of nerve [54], breast [55], prostate [56] and the gastrointestinal tract [57].

The ImFISH technique, as originally described, combined conventional double immunofluorescence, using fluorochrome-conjugated secondary antibodies, with a standard FISH technique and its interpretation was done directly on a fluorescence microscope [51].

As part of our ongoing efforts to improve the diagnostic yield and accuracy of smaller and less cellular brain tumor samples, we decided to try the ImFISH technique on oligodendroglial tumors. We began by looking at a combination of FISH and MIB-1 immunostaining since the proliferation index, as measured with the MIB-1 antibody, is routinely reported on most brain neoplasms and the chromosome 1p and 19q status is routinely determined for all oligodendroglial tumors in our laboratory. It thus seemed appropriate to us to combine these two techniques into one.

Preliminary tests by immunofluorescence led to inconclusive results, especially in tumors with a low proliferative index, in which the identification of neoplastic cells proved difficult. Autofluorescence of erythrocyte clusters was a problem, as was distinguishing proliferating blood vessels from adjacent tumor cells, which made the analysis time consuming and difficult to reproduce. Therefore we decided to replace the immunofluorescence step with a conventional chromogenic immunohistochemical technique. Although this modification meant that the analysis could no longer be performed solely by fluorescent microscopy, we were able to use a standard digital microscopy setup to capture both fluorescent and bright-field microscopic images and superimpose the images.

The results of our preliminary assays encouraged us to extend this technique to a series of 36 oligodendroglial tumors previously analyzed in our laboratory by standard FISH technique.

To validate this technique we first studied the ImFISH reproducibility between two independent observers.

Results obtained by ImFISH technique were then compared with those obtained by standard FISH technique which served as the reference in our study.

To determine the reliability of this technique on a limited number of cells, we analyzed a decreasing number of cells (60 cells, 40 cells and 20 cells) by ImFISH and checked if the reported 1p and 19q status remained constant or not.

In our series, chromosomal data were also compared with the clinical and surgical data, and with MIB-1 labeling indices to seek any correlation existing between these different parameters.

For all our analysis, we decided to calculate chromosomal status using both the combination and ratio methods, in order to permit the widest possible comparison with the literature data.

## Materials and Methods

### Ethics statement

The Research Ethics Committee of the Centre Hospitalier Universitaire de Québec was consulted for this study and decided that its approval was not necessary. The committee waived the need for consent, the aim of this study being the optimization of an institutional diagnostic technique with anonymized data.

### Tissue samples

Formalin fixed paraffin-embedded (FFPE) tissue from 36 brain tumor samples (biopsies or surgical resections) with previously established 1p/19q status by FISH was pre-selected for the ImFISH study. All tumors were classified and graded according to the guidelines of the World Health Organization [58] by two neuropathologists (PVG and SS). The cases included 11 WHO grade II oligodendrogliomas (OII), 22 WHO grade III anaplastic oligodendrogliomas (OIII) and 3 WHO grade IV glioblastomas with oligodendroglioma component (GBMO). FISH analysis of 1p/19q was initiated in all cases during diagnostic work-up.

### FISH

FISH analysis was performed using the LSI 1p36/19q13 Dual-Color Probe kit (Abbott Molecular Inc., Abbott Park, Illinois, USA). Slides were immersed in 0.2 N HCl for 20 minutes at room temperature, rinsed with purified water and incubated in pretreatment solution (2x saline sodium citrate (SSC) for 30 minutes at 97°C. After washes with purified water and 2x SSC, pretreated slides were digested with a pepsin solution (Dako) at 37°C for 5 minutes, rinsed in 2× SSC at RT for 5 minutes, and dehydrated using graded ethanol (70%, 85%, and 100% for 1 minutes each) and finally air dried. The probe mix (5 to 15 µl) was added to each slide according to the manufacturer's instructions and the hybridization area was covered with a coverslip then sealed with rubber cement. Target DNA and probes were codenatured at 74°C for 5 minutes and incubated at 37°C overnight in a humidified hybridization chamber (ThermoBrite, Abbott Molecular Inc.). Posthybridization washes were performed in NP40 0.3%/2×SSC (pH 7) at 75°C for 2 minutes. Finally, the slides were air dried and counterstained with DAPI (4′,6-diamidino-2-phenylindole) diluted in Vectashield (Vector, Burlingame, CA, USA). Signal acquisition was performed for each slide over 10 to 15 more representative areas. These areas were automatically captured at x400 using a Metasystems station (Zeiss MetaSystems, Thornwood, NY) equipped with a Zeiss Axioplan fluorescent microscope.

FISH interpretation for 1p and 19q status using the combination method was performed according to the guidelines of the International Society of Paediatric Oncology (E-SIOP Neuroblastoma Study Group) for studies of peripheral neuroblastic tumours [43]. For each case, two independent and blinded observers (FS and SS) assessed 100 non-overlapping nuclei for red ‘R’ (marker) and green ‘G’ (reference) signals. The frequencies of signal patterns for 1p (R) and 1q (G) on one slide, and for 19p (G) and 19q (R) on another slide, were noted. The cut-off of nuclei that had to show deletion was calculated on a series of 10 non-neoplastic brain tissue samples (from epilepsy surgery cases and autopsy brains). This cut-off was calculated using mean +3 SD and was set at 50% for both 1p and 19q. Cases above the cutoff were considered deleted and those under the cut off were considered normal or imbalanced according to the literature guidelines [43], [44].

For each case the signal ratio of red signals to green signals per cell was also established. A ratio ≤0.8 was considered to indicate a deletion whereas a ratio between 0.8 and 1.1 was considered to indicate a normal status on the chromosomal arm. A ratio over 1.1 was considered to indicate polysomy and was classified in the imbalanced status subgroup [2], [30].

### ImmunoFISH

**Figure 1:**
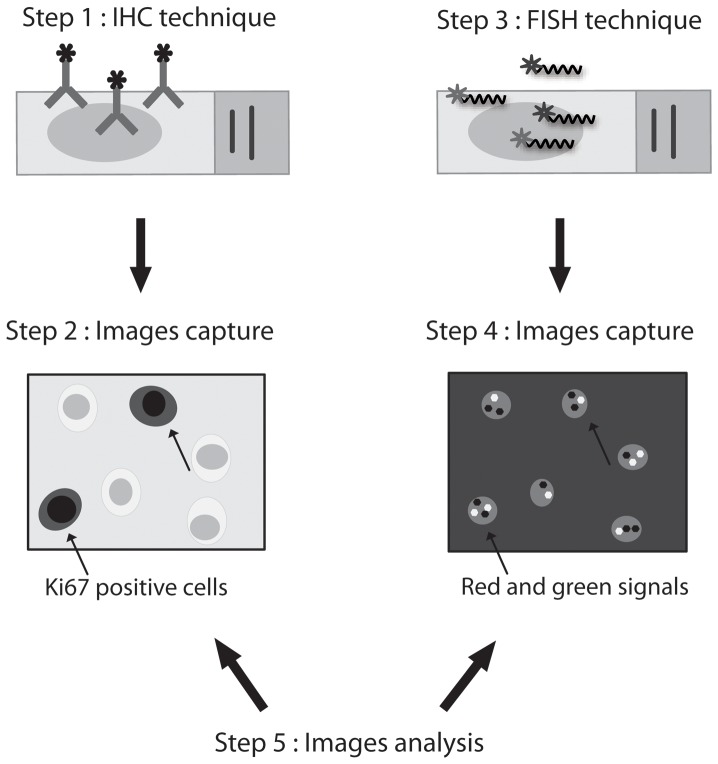
Overview of ImmunoFish procedure. In the first step, immunohistochemistry with MIB-1 antibody is performed on FFPE oligodendrogliomas. Digital images of the 10 most labelled areas are taken at high magnification (x 400). Then the slide is washed and used for a second FISH step using 1p36 or 19q13 probes. Digital images of the same 10 areas that were selected based on MIB-1 labelling are taken at the same magnification. Analysis of the two sets of images is done simultaneously on two separate screens. Only cells with MIB-1 labelling are taken into account for FISH analysis.

Immunohistochemistry (IHC) procedures were performed on 4-µm-thick sections obtained from FFPE brain tumor tissues. Sections were deparaffinized in 2 xylene washes for 5 min and rehydrated in 100% ethanol baths. Antigen retrieval was then carried out in a PT Link pretreatment system (Dako, Mississauga, Ontario, Canada). After rinsing the slides in purified water and in wash buffer (PBS-Tween 20x, Dako), the sections were incubated in a humid chamber at room temperature (RT) with monoclonal mouse primary antibody against Ki67 (MIB-1; Beckman Coulter, Fullerton, CA, USA) diluted 1∶100 in EnVision Flex antibody diluent (Dako) for 15 minutes. Immunolabelling was revealed with the EnVision G2/AP System with permanent red chromogen (Dako) according to the manufacturer's instructions except that all incubations were performed at room temperature for 15 min. The sections were then counterstained with hematoxylin. Tumor cells with red staining were considered positive, regardless of IHC staining intensity. MIB-1 signal acquisition was performed for each slide over 15 to 30 areas that were selected by an observer (CD). These areas were automatically captured at x400 using the same microscope and image analysis software than previously described for the FISH (Figure 1). After MIB-1 signal acquisition, slides were washed in a 100% ethanol bath to remove the red aminoethylcarbazol (AEC) chromogen staining from tissues then air dried. FISH analysis was performed on the same slide using the same procedure as described above. Fluorescent signals were automatically recorded at x400 in the same preregistered positions as for MIB-1 signal acquisitions (Figure 1).

IHC and FISH images from the ImFISH technique were analysed simultaneously on two separate computers screens (Figure 1) by two independent and blinded observers (CD and SS).

The MIB-1 labelling index (LI) was calculated for each case on IHC images by counting 100 contiguous cells in the most positive areas.

For each probe 1p and 19q, a total of 60 MIB-1 positive nuclei were analyzed for the chromosomal status using the same procedures previously described for FISH. For the combination method, and in the absence of positive immunostaining in our non-tumor brain control series, we decided to set our cut-off at the median value of our tumor series which corresponds to a value of 65% for both 1p and 19q. For the ratio method, established values were the same as for the FISH.

### Statistical analyses

All statistical analyses were carried out with the R statistical environment (http://www.R-project.org/). Inter-observer agreement of ImFISH analysis was estimated by calculating Cohen's kappa coefficient (κ) with the Kappa function of the R package *vcd*. We considered a κ value between 0.6 and 0.8, as good agreement and a value >0.8 as high concordance.

ImFISH reliability was studied by calculation of the sensitivity and the specificity of the method compared to FISH independently for each of the two observers' values.

Chi-square test was performed for group comparisons between FISH and ImFISH analysis for 20 cells, 40 cells and 60 cells. A significant correlation between two parameters was noted at the 95% confidence interval and a P value <0.05 was considered as a significant difference between two groups.

Mib1 labelling indices, histologic and clinical data were compared with each other according to the chromosomal status using logistic regression models. P values ≤0.05 were considered as statistically significant.

## Results

### Patient characteristics

Our 36 patients included 22 females and 14 males (respectively 60% and 40% of the cohort). The age of patients ranged from 26 to 82 years with a median age of 55 years, and 14 patients were younger than 50 years.

The patients underwent open surgery with gross total or partial tumor resection (n = 26; 72%) or stereotactic biopsies (n = 10; 28%).

For statistical analysis purposes tumor location was considered to be the lobe of the brain within which the largest volume of the glioma resided if more than one lobe was affected. In our series, tumor locations in order of decreasing frequency were frontal (n = 20; 55%), temporal (n = 10; 28%), parietal (n = 5; 14%) and insular lobe (n = 1; 3%).

Six cases among the 36 cases were recurrent tumors (17%).

### Interpretation of ImFISH results

**Figure 2:**
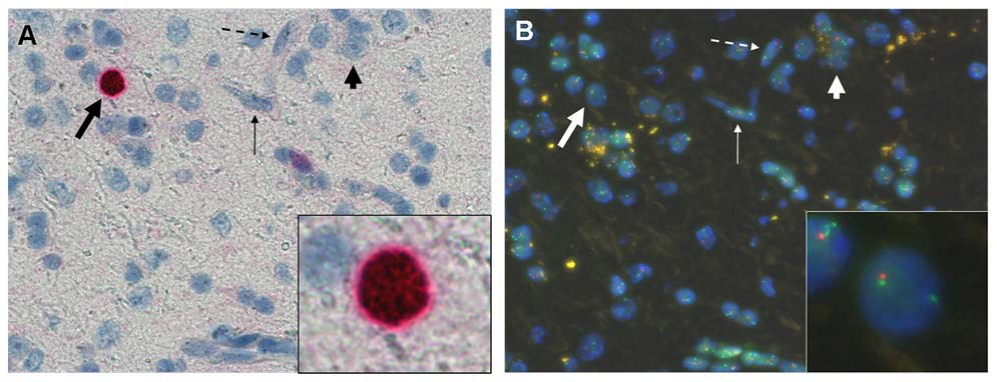
ImFISH interpretation. ImFISH technique allows a simultaneous analysis of nuclear staining with MIB-1 antibody by conventional immunohistochemistry (A) and *in situ* hybridization with chromosomal 1p and 19q probes (B) on two separate screens. The light haematoxylin counterstaining of the immunohistochemistry step allows an easy identification of the majority of the cells analyzed: oligodendrocytes (thick arrows), astrocytes (thin arrows), neurons (short arrows) and endothelial cells (dotted arrows). Only MIB-1 labeled nuclei with an oligodendroglial morphology are analysed by FISH (framed nuclei on A and B).

For a given sample area, the ImFISH technique generates two distinct images, MIB-1 IHC and 1p or 19q FISH, that are easily interpreted by the user. These images are easily stackable by image software for analysis together on the same screen or can be analyzed separately on two separate screens (Figure 2). We preferred the second solution for this study because of its simplicity, knowing that many people who examine chromosomal status by FISH do not necessarily use sophisticated image processing and image fusion software.

A phosphatase alkaline system using an AEC chromogen proved superior to the widely-used peroxidase system with its brown diaminobenzidine (DAB) chromogen. DAB chromogen remained adherent to the target despite successive washes causing non specific fluorescence and difficulties of interpretation during the FISH analysis, whereas AEC chromogen was very easy to remove with an ethanol bath.

After a short time to adapt to the two screen method used, the user can easily identify the positive nuclei on immunohistochemical labeling images and their counterparts on FISH images. An average of 15 minutes per case is necessary to analyze 60 cells, which is not much more longer than the time required in routine practice to count 100 to 200 non-overlapping cells by standard FISH technique in our experience at our institution.

The immunohistochemical counter-staining by hematoxylin in the MIB-1 IHC allows a better histological quality control of selected area than those disclosed by DAPI on FISH images. This allows a more detailed morphological analysis of the cell nuclei, and an easier identification of oligodendrocytes with their classical rounded nuclei (Figure 2, thick arrow); astrocytes displaying usually an elongated nuclei (Figure 2, thin arrow) and neurons displaying a prominent nucleoli (Figure 2, short arrow).

In anaplastic oligodendrogliomas and GBMO, nuclear MIB-1 labeling can be observed in neoplastic cells but also in some histiocytes associated with necrotic areas and some proliferating endothelial cells. To avoid these false positives in our study, we decided to exclude peri-necrotic areas from our analysis to prevent inadvertent inclusion of macrophages. Proliferating endothelial cells, for their part, were easily identified on counterstain and have been excluded from MIB-1 and FISH analysis (Figure 2, dotted arrow).

The sharpness of the nuclear staining with MIB-1 antibody facilitated nuclear identification and delineation even in the case of overlapping nuclear profiles, allowing the assessment of chromosome status for these cells which are usually excluded from analysis with standard FISH.

### Inter-observer reliability of ImFISH technique

The inter-observer reliability of ImFISH was calculated by the Kappa score.

A good concordance was observed between the two readers by the ratio method for 1p (κ = 0.71) and 19q (κ = 0.72). These results are very similar to our internal institutional control values for the FISH technique. A higher concordance was observed by the combination method for both 1p (κ = 0.86) and 19q (κ = 0.95).

### Comparison between FISH and ImFISH techniques

FISH yielded interpretable results in 100% of cases for 1p and 19q (Table 1). Chromosome 1p/19q alterations included both deletions (1p: n = 22 and 19q: n = 25 respectively 60% and 69%) and imbalances (1p: n = 9 and 19q: n = 8 respectively 25% and 22%). Combined 1p/19q deletion was detected in 22 cases (60%). Solitary 1p imbalance was noted in 2 cases (6%) and solitary 19q loss was noted in 3 cases (8%). In our series, no case showed 1p deletion alone. One case was imbalanced for both 1p and 19q. A minority of cases showed no abnormality (1p: n = 5 and19q: n = 3 respectively 14% and 8%).

**Table 1:** Histological grade, MIB1 labelling Index (LI) and 1p/19q ImmunoFISH and FISH status, by ratio and combination methods.

Patient #	WHO Grade	MIB1 LI (%)	Ratio								Combination %															
			1p		Status		19q		Status		1p						Status		19q						Status	
			Obs. #1	Obs. #2	ImFISH	FISH	Obs. #1	Obs. #2	ImFISH	FISH	obs. #1			obs. #2					obs. #1			obs. #2				
											D	N	I	D	N	I	ImFISH	FISH	D	N	I	D	N	I	ImFISH	FISH
13	II	3	1.02	1.14	**N/I**	**N**	1.02	1.02	**N**	**N**	20	55	25	15	40	45	**N**	**N**	15	70	15	15	50	35	**N**	**I** *
33	II	5	0.44	0.47	**D**	**D**	0.42	0.49	**D**	**D**	92	3	5	88	5	7	**D**	**D**	98	2	0	88	7	5	**D**	**D**
16	II	6	0.38	0.68	**D**	**D**	0.48	0.54	**D**	**D**	75	15	10	62	18	20	**D**	**D**	80	15	5	70	17	13	**D**	**D**
23	II	6	1.13	1.16	**I**	**N** *	0.81	0.95	**N**	**D** *	12	57	31	22	35	43	**N/I**	**N**	37	52	11	28	47	25	**N**	**D** *
24	II	6	0.44	0.60	**D**	**D**	0.46	0.51	**D**	**D**	90	8	2	82	7	12	**D**	**D**	82	10	8	90	5	5	**D**	**D**
31	II	6	0.43	0.55	**D**	**D**	0.5	0.67	**D**	**D**	87	12	1	80	10	10	**D**	**D**	80	13	7	67	12	22	**D**	**D**
6	II	7	0.8	0.79	**N**	**N**	0.93	0.87	**N**	**N**	38	23	39	43	13	43	**I**	**I**	13	60	27	27	48	25	**N**	**I** *
14	II	9	0.81	0.84	**N**	**N**	0.88	0.87	**N**	**N**	46	28	26	43	22	35	**I**	**I**	23	37	40	28	17	55	**I**	**I**
30	II	9	0.64	0.93	**D/N**	**D**	0.82	0.74	**D**	**D**	28	49	23	30	40	30	**N**	**D** *	32	51	17	32	47	22	**N**	**D** *
28	II	10	0.54	0.63	**D**	**D**	0.54	0.58	**D**	**D**	73	10	17	62	12	27	**D**	**D**	67	18	15	65	12	23	**D**	**D**
20	II	11	0.55	0.53	**D**	**D**	0.53	0.51	**D**	**D**	83	10	7	83	7	10	**D**	**D**	83	12	5	88	7	5	**D**	**D**
9	III	5	1	0.94	**N**	**N**	0.98	1.11	**N**	**N**	13	58	29	29	38	32	**N**	**I** *	23	59	18	20	43	37	**N**	**I** *
27	III	5	0.9	1.20	**N/I**	**D**	0.95	0.93	**N**	**D**	30	60	10	22	39	39	**N**	**D** *	10	76	14	20	57	23	**N**	**D** *
18	III	6	0.99	1.09	**N**	**I** *	1.02	1.11	**N/I**	**I**	22	57	21	18	43	38	**N**	**I** *	20	59	21	13	63	23	**N**	**I** *
25	III	8	0.48	0.58	**D**	**D**	0.44	0.50	**D**	**D**	80	10	10	75	8	17	**D**	**D**	75	8	17	80	8	12	**D**	**D**
1	III	10	0.55	0.62	**D**	**D**	0.58	0.53	**D**	**D**	67	13	20	60	3	37	**D**	**D**	68	15	17	65	3	32	**D**	**D**
12	III	11	0.99	1.01	**N**	**D** *	0.96	0.97	**N**	**N**	8	69	23	20	47	33	**N**	**I** *	12	48	40	7	77	17	**N**	**N**
4	III	12	1.01	1.05	**N**	**N**	0.43	0.43	**D**	**D**	8	65	27	10	47	43	**N**	**N**	85	12	3	85	12	3	**D**	**D**
11	III	12	1.19	1.26	**I**	**N**	0.95	1.01	**N**	**N**	8	50	42	10	37	53	**I**	**I**	12	57	31	17	40	43	**N**	**N**
26	III	13	0.5	0.54	**D**	**D**	0.43	0.55	**D**	**D**	88	5	7	92	2	7	**D**	**D**	87	7	6	80	8	12	**D**	**D**
34	III	13	0.49	0.46	**D**	**D**	0.49	0.59	**D**	**D**	90	3	7	92	3	5	**D**	**D**	88	7	5	77	15	8	**D**	**D**
21	III	16	0.49	0.56	**D**	**D**	0.5	0.55	**D**	**D**	87	8	5	78	15	7	**D**	**D**	87	6	7	75	13	12	**D**	**D**
32	III	20	0.518	0.61	**D**	**D**	0.53	0.53	**D**	**D**	67	5	28	52	10	38	**D**	**D**	83	13	4	80	12	8	**D**	**D**
2	III	21	0.52	0.50	**D**	**D**	0.52	0.49	**D**	**D**	83	7	10	83	3	13	**D**	**D**	82	12	6	87	8	5	**D**	**D**
17	III	21	0.5	0.49	**D**	**D**	0.58	0.54	**D**	**D**	75	7	18	65	5	30	**D**	**D**	53	15	32	58	7	35	**D**	**D**
5	III	22	0.57	0.49	**D**	**D**	0.52	0.57	**D**	**D**	73	10	17	75	12	13	**D**	**D**	68	10	22	65	13	22	**D**	**D**
8	III	22	1.1	1.03	**N**	**N**	1.03	1.17	**N/I**	**N**	22	52	26	15	25	60	**N/I**	**N**	17	53	30	13	40	47	**N**	**N**
36	III	23	0.52	0.54	**D**	**D**	0.45	0.57	**D**	**D**	65	15	20	59	13	28	**D**	**D**	78	7	15	60	17	23	**D**	**D**
7	III	26	1.23	1.47	**I**	**N** *	1.05	1.01	**N**	**N**	8	32	60	17	28	55	**I**	**I**	1	47	52	15	22	63	**I**	**I**
35	III	29	0.33	0.38	**D**	**D**	0.38	0.42	**D**	**D**	94	3	3	93	3	3	**D**	**D**	83	7	10	87	2	12	**D**	**D**
15	III	34	0.84	0.89	**N**	**N**	0.65	0.70	**D**	**D**	37	37	26	28	22	50	**I**	**I**	57	28	15	42	15	43	**I**	**I**
22	III	34	0.57	0.51	**D**	**D**	0.45	0.60	**D**	**D**	74	13	13	75	3	22	**D**	**D**	78	5	17	53	15	32	**D**	**D**
29	III	34	0.52	0.53	**D**	**D**	0.45	0.59	**D**	**D**	65	10	25	60	17	23	**D**	**D**	77	5	18	65	10	25	**D**	**D**
10	IV	14	1.37	1.27	**I**	**N** *	1.03	1.09	**N**	**N**	8	43	49	17	37	47	**I**	**I**	13	54	33	12	33	55	**N/I**	**I**
19	IV	14	0.51	0.62	**D**	**D**	0.55	0.51	**D**	**D**	70	22	8	73	20	7	**D**	**D**	67	20	13	72	15	13	**D**	**D**
3	IV	19	1.05	1.16	**N/I**	**N**	0.54	0.49	**D**	**D**	18	47	35	13	37	50	**N**	**N**	83	10	7	92	3	5	**D**	**D**

**D**: deletion, **N**: no deletion and no imbalance, **I**: imbalance

*ImFISH/FISH discordance

ImFISH was calculated on 60 nuclei and FISH on 100.

ImFISH also yielded interpretable results in 100% of cases for 1p and 19q (Table 1) however in 4 out of 72 tests (36 for 1p and 36 for 19q) the technique failed to identify 60 MIB-1 positive cells as initially planned (mean∶31 nuclei; min∶20 – max∶47). Chromosome 1p/19q alterations included both deletions (1p: n = 20 and 19q: n = 22 respectively 55% and 60%) and imbalances (1p: n = 6 and 19q: n = 3 respectively 17% and 8%). Combined 1p/19q deletion was detected in 20 cases (55%). Solitary 1p imbalance was noted in 3 cases (8%) and solitary 19q loss was noted in 2 cases (6%). No case showed 1p deletion alone and one case was imbalanced for both 1p and 19q. A minority of cases showed no abnormality (1p: n = 10 and 19q: n = 11 respectively 28% and 31%).

Compared to FISH, the ImFISH exhibits a very high sensitivity (100%) and a high specificity (91%) for 1p and/or 19q deleted cases. Combination and ratio methods give nearly identical results (Table 2).

**Table 2:** Sensitivity and specificity of ImFISH according to the FISH.

	Combination			Ratio		
	D	N	I	D	N	I
**Sensitivity 1p**	1	0.84	0.94	0.96	0.87	0.82
**Specificity 1p**	0.91	0.7	0.67	0.89	0.5	0
**Sensitivity 19q**	1	0.77	1	1	0.89	0.95
**Specificity 19q**	0.88	1	0.44	0.9	0.83	0.5

Sensitivity and specificity was calculated for each of the 2 observers and for each of the chromosomal analysis method (combination and ratio) on 36 cases. The sum of both results is reported here (total n = 72).

The sensitivity of the technique decreases slightly for normal cases (Combination∶84%–Ratio∶87%) for both 1p and 19q. The specificity also decreases slightly for 19q normal status (Combination∶100%–Ratio∶83%) but much more for 1p normal status (Combination∶70%–Ratio∶50%).

For imbalanced cases the sensitivity remains high for both 1p (∼90%) and 19q (∼100%) imbalances regardless of the method of calculation used. Nevertheless the specificity decreases strongly to roughly 50% for both 1p and 19q imbalances (Table 2).

### ImFISH results according to the number of cells analyzed

Study of the minimal number of cells needed for ImFISH analysis was made on three decreasing series of cells (60 cells, 40 cells and 20 cells). The final result of each of these series was compared to the results initially obtained by FISH (Table 3). This study was performed on 34 of our 36 cases; in two cases ImFISH failed to label at least 60 cells. Results were divided into subgroups of deleted, normal and imbalanced status and did not show a statistical difference by the Chi-square test between the cohorts of the different subgroups obtained by FISH and ImFISH technique. Likewise there were no significant difference between FISH and ImFISH results calculated on 60, 40 or 20 cells and this regardless of the method of calculation used (combination or ratio) or the type of chromosomal status studied (Chi-square test not significant). The results from both observers were very similar; hence we have shown the results of only one observer in Table 3.

**Table 3:** Comparison between FISH and ImFISH results.

	Combination		Ratio
	FISH	ImFISH 20	ImFISH 40	ImFISH 60	FISH	ImFISH 20	ImFISH 40	ImFISH 60
Codeletion	21	17	18	20	21	22	21	20
Deletion 1p	0	1	1	1	1	1	1	1
Deletion 19q	3	4	3	2	4	3	3	4
Normal 1p	5	7	8	6	11	5	7	8
Normal 19q	3	8	8	8	8	9	9	9
Imbalancement 1p	8	9	7	7	1	6	5	5
Imbalancement 19q	7	5	5	4	1	0	1	1
Chi-square test		NS	NS	NS		NS	NS	NS

NS  =  non significant.

Number of final diagnoses obtained by FISH and ImFISH on 20, 40 and 60 cells respectively and for each combination and ratio chromosomal analysis method. Chi-square test was applied between FISH and each of three ImFISH trials.

It should be noted that our results show that the calculation method by combination or ratio does not always produce the same result, especially for normal and imbalanced chromosome status (Table 1 and 3).

### Comparison of chromosomal status with clinical, surgical and immunohistochemical data

Loss of 1p and 19q was observed in 6 (of 11) (55%) of OII, 13 (of 22) (59%) of OIII and 1 (of 3) (33%) of GBMO.

This codeletion was observed in 16 (of 30) (53%) of primary tumors and 4 (of 6) (66%) of recurrent tumors.

The frequency of 1p/19q alterations was not significantly different in WHO Grade II, Grade III or Grade IV tumors or in primary and recurrent tumors.

No correlation was observed between the sex or the age of the population and the frequency of the 1p/19q status.

Loss of 1p and 19q was identified in 15 (of 20) (75%) of frontal, 2 (of 10) (20%) of temporal, 2 (of 5) (40%) of parietal and 1 (of 1) (100%) of insular located tumors. This repartition was statistically significant (p<0.05). When analyzed separately only 1p deletion remains significantly associated with the cerebral location: 75% of the 1p deleted tumors are located in the frontal lobe (p = 0.01) and 50% of the 1p normal status tumors are located in the temporal lobe (p = 0.01).

In our series MIB-1 immunolabelling appeared as nuclear staining (Figure 1). MIB-1 mean indices ranged from 7% for OII (min: 3 – max: 11), 18% for OIII (min: 5 – max: 34) and 16% for GBMO (min: 14 – max: 19). These proliferative index values was statistically significant between the low grade and the high grade tumors (p<0.01).

Logistic regression analysis observed no significant correlation between the MIB-1 labelling index (LI) and the 1p and/or the 19q deletion.

## Discussion

Our modified ImFISH technique is easy to perform and to analyse, requiring a short time to learn. In the absence of clearly established guidelines for ImFISH use in the literature, we favoured an unbiased approach performing a double independent observer analysis for each step in our study, and using both commonly used methods of FISH chromosomal status reporting∶i.e. combination and ratio.

The Kappa test shows a good concordance between the results for both observers. This concordance is higher for the combination method than the ratio method. These results may seem surprising given that our cut off for the combination method was established theoretically. However, our results highlight the fact that the two common methods for calculating the chromosomal status, although convergent in most cases are not necessarily superimposable. The combination method is more adapted to detect small groups of cells with a different chromosomal profile while the ratio method gives an overall average of all examined cells and appears less appropriate to highlight a particular chromosomal profile from a small group of cells. In the literature, the use of one method or the other to determine the chromosomal status of 1p and 19q is a matter of institutional practice and there is no clear evidence that it is better to use of one method or another.

ImFISH inter-observer results are equivalent or superior to those accepted for the FISH in our internal institutional practice. This emphasizes the benefits of the ImFISH when focusing the cellular analysis on a limited group of pre-selected cells (here MIB-1 immunoreactive cells) which reduces the field of analysis for the observer and increases greatly the probability that two independent observers will analyze the same cells.

In this series, the sensitivity of ImFISH compared to FISH is high regardless of the method of calculation used (combination or ratio) (Table 2). This confirms the value of this technique in identifying true positive cases while avoiding false negatives. Sensitivity appears satisfactory for deleted and normal cases but inadequate for imbalanced cases. Some cases are imbalanced by FISH and normal by ImFISH and vice versa, regardless of the method of calculation used (Table 1). This discrepancy is important to study further given the growing awareness of importance of the 1p/19q imbalanced population in the prognosis of oligodendroglial tumours. Recent studies show a strong correlation between the presence of an imbalanced tumor cell population and a shorter overall survival [12], [21]. Analysis of imbalanced cases by FISH is known to pose problems of interpretation due to chromosomal polyploidy and multiple probe signals [23]. In our series, changing the cut-off values used for ImFISH or extending the analysis to 100 MIB-1 labelled tumor cells instead of 60 did not eliminate the discrepancy (data not shown), suggesting a real difference in cell analysis by these two techniques, since only the MIB-1 immunoreactive cells are analyzed by ImFISH whereas in FISH cell selection is independent of cell type or cell cycle status. This difference may explain the selection by ImFISH of many more potentially false positive cells than by FISH, thereby decreasing the value of its specificity without jeopardizing its sensitivity. We feel that these potentially false positive cells are most likely genetically unstable true-positive cells not taken into account by the standard FISH technique. The present study contains few imbalanced cases, so analysis of a larger cohort would be necessary to verify these results. Other molecular analysis techniques such as LOH analysis or CGH may also be of interest to characterize the discordant cases between FISH and ImFISH. These techniques are efficient in highly cellular tumor cases since at least 70% of the cells need to be part of the tumor for a satisfactory interpretation, but they are not appropriate for tumors of low cellularity for which FISH and ImFISH are more suitable [35], [37]. For tumors of low cellularity, a correlation with overall survival data would be necessary to determine which imbalanced cases by FISH or ImFISH are technical true positives but biological false negatives.

In our study, ImFISH technique gives substantially similar results to FISH even having analyzed 60, 40 or just 20 cells. These results were expected for deleted cases because these latter cases are usually very straightforward and easily analyzable even on a small number of cells [26], [45]. To our surprise, the results of normal and imbalanced cases, although less similar, remain statistically significant and underline the interest of the ImFISH. Unfortunately, for normal and imbalanced cases, the FISH literature does not offer clear quantitative data allowing us to more detailed comparisons between these two techniques.

Nevertheless, our results highlight the benefits of practicing ImFISH on tumor samples of low cellularity (diffuse low grade gliomas, periphery of the tumor or biopsy material) since the analysis of 20 cells still allows to provide a satisfactory result on 1p and 19q chromosomal status (Table 3).

Despite the preselected character of our series, the majority of our cases were codeleted as typically described in the literature [13], [17], [22], [30], [59] and there was no chromosomal abnormalities differences between the primary and the recurrent tumoral population underlining the genetic stability of the majority of recurrent oligodendrogliomas [23].

In our series, there was a significant association between frontal lobe location and the allelic loss of chromosomes 1p and 19q and between temporal lobe location and maintenance of 1p/19q status as described in many studies of the literature [1], [18], [60], [61]. These findings suggest that molecular subsets of oligodendroglial tumors might arise from site-specific precursor cells and arise preferentially in certain lobes, with tumors having deletion on 1p and 19q occurring most frequently in the nontemporal lobes [34], [60], [62].

Finally MIB-1 LI appears useful for the discrimination between low and high grade oligodendroglioma but did not show a significant association with 1p19q deletion status in concordance with the literature [34], [58], [63], [64]. These results suggest that the neoplastic protective effect observed in 1p19q deleted oligodendrogliomas does not involve the cell cycle mechanism.

## Conclusion

The ImFISH technique has never been applied before in the study of gliomas to our knowledge. The technique described in this study is easy to perform and analyze and does not require additional technical equipment or interpretation expertise to be achieved. It is reproducible and generates similar results to the classic FISH technique. It appears best suited to low density tumoral areas or poorly cellular tumoral samples (low grade gliomas, peritumoral area or biopsy material).

The present study lacks a correlation between molecular markers and survival times because the length of follow-up for our patient cohort is too short for meaningful statistical analysis. Future studies are planned to broaden the application of this technique which offers many advantages for the study of pure or mixed oligodendrogliomas and other gliomas. Among the possibilities we note the interest to combine immunohistochemical analysis of other known prognostic factors in gliomas such as TP53, MGMT, EGFR and IDH1 expression with the chromosome 1p and 19q status within a given sub-population of tumor cells [9], [19], [34], [64], [65], [66]. Other oligodendroglial chromosomal abnormalities such as gain or loss of chromosome 10 or 7 could also been studied by this technique [64], [67], [68].

Finally ImFISH analysis can be easily automated, which may lead to easier validation of automated image analysis software for FISH within each institution. By focusing analysis on a limited number of pre-selected cells, ImFISH removes a potential source of inter-observer variability whether a human or computer examines the cells.
